# Practical Insights from Operationalizing a Remote Symptom Monitoring Program Using a Commercial Platform in a Canadian Community Cancer Clinic: A Post-Implementation Qualitative Evaluation

**DOI:** 10.3390/curroncol33090561

**Published:** 2026-09-16

**Authors:** Melissa J. Chao, Eric Liow, Nasia A. Sheikh, Jeremy Ho

**Affiliations:** 1 Vancouver Coastal Health, 7000 Westminster Hwy, Richmond, BC V6X 1A2, Canada; melissa.chao@torontomu.ca; 2 School of Medicine, Toronto Metropolitan University, 150 Central Park Drive, Brampton, ON L6T 2T9, Canada; 3 Vancouver Coastal Health Research Institute, 2635 Laurel Street, Vancouver, BC V5Z 1M9, Canada

**Keywords:** remote symptom monitoring, electronic patient-reported outcomes, cancer care implementation, workflow integration, qualitative thematic analysis

## Abstract

Remote symptom monitoring using electronic patient-reported outcomes reduces symptom burden and acute care use in oncology, but most programs have been established in academic centers or funded trials using purpose-built software. We examined how the REmote Symptom and Patient mONitoring System (RESPONSe) was implemented and subsequently funded as a recurrent clinical service at a Canadian community cancer clinic using a configured commercial platform, patients’ own devices, and no formal institutional mandate. We conducted a qualitative post-implementation process evaluation at Richmond Cancer Clinic, British Columbia, reported following the Consolidated Criteria for Reporting Qualitative Research. Eight staff, purposively sampled by role and phase of involvement, were invited and participated, which consisted of one oncologist, three nurses, two virtual health staff, and two administrators. Interviews were analyzed using deductive–inductive thematic analysis. Three themes were identified: tailoring technology for clinical fit; designing care around patient and clinician users, centered on a dedicated symptom management nurse; and managing up towards operationalization. Operationalization was achievable using a customizable commercial platform, but depended on the interaction of iterative technology adaptation, dedicated clinical ownership, evolving care workflows, and sustained organizational advocacy. We propose five transferable implementation strategies mapped to established implementation frameworks.

## Simple Summary

People undergoing chemotherapy often experience difficult side effects between clinic visits and must judge for themselves when to contact their cancer team for help. Programs where patients report symptoms regularly by phone or computer, with a nurse following up, improve care and reduce emergency visits. Most of these programs have been set up in large hospitals or as funded research studies, so little is known about whether smaller community clinics can run them. We interviewed eight staff involved in building and running such a program at a community cancer clinic in Richmond, British Columbia. They described the work needed to adapt commercial software for cancer care, to staff the program, and to keep hospital leaders engaged and funding in place. Because about half of systemic therapy in British Columbia is given in community clinics, these lessons may help other smaller clinics start similar programs without large budgets or specialized technology.

## 1. Introduction

Cancer remains a leading cause of morbidity and mortality worldwide, with an estimated 20.6 million new cancer cases and 9.8 million cancer-related deaths occurring globally in 2024 [1]. As advances in cancer detection and treatment continue to improve survival, increasing attention has shifted toward improving the quality and safety of cancer care treatment. Symptom burden of chemotherapy, a cornerstone of cancer treatment, is a central concern in oncology, as patients commonly experience distressing physical and psychological symptoms that negatively affect daily functioning, quality of life, treatment adherence, and healthcare utilization [2,3]. Between clinic visits, patients are often expected to independently recognize concerning symptoms and determine when to contact their cancer team, contributing to delays in symptom identification and management. Chemotherapy-related side effects have consequently been identified as major drivers of potentially preventable emergency department (ED) visits [4,5]. These challenges underscore the need for more effective strategies to monitor symptoms between visits and provide timely support.

Recognizing that symptoms are often under-recognized during routine oncology care, cancer symptom science has increasingly emphasized the importance of systematic symptom assessment using patient-reported outcomes. A promising approach that has emerged in addressing these challenges is the use of electronic patient-reported outcomes (ePROs). Recently, ePROs have demonstrated significant benefit in the outpatient management of patients with cancer [6,7,8,9,10]. Integration of ePROs into routine care enables symptom monitoring between visits, supporting earlier recognition of treatment-related toxicities and more timely clinical intervention. Prior studies have shown that ePRO-based remote symptom monitoring (RSM) improves patient-provider communication, enhances patient-reported quality of life, and reduces acute care utilization, including ED visits and hospitalizations; in some settings, it has also been associated with improved survival [6,7,8,9,10,11,12,13].

Reflecting this evidence, professional organizations including the American Society of Clinical Oncology (ASCO) have recommended routine incorporation of patient-reported outcomes into clinical practice [11]. However, real-world adoption in oncology settings remains limited due to several logistical challenges, such as workflow integration, clinical capacity and applicable technology, leaving a persistent gap between demonstrated efficacy and routine practice [6,10]. While the effectiveness of ePRO-based remote symptom monitoring has been well established, fewer studies have examined how these programs can be successfully embedded into routine clinical workflows and sustained beyond pilot implementation [11]. Understanding the organizational, operational, and contextual factors that influence implementation is critical to informing broader scale-up across cancer programs.

The Richmond Cancer Clinic designed, implemented and operationalized the REmote Symptom and Patient mONitoring System (RESPONSe) Program, a RSM program that utilizes ePROs for patients initiating outpatient chemotherapy [14,15]. The program customized a commercially available digital platform to support structured symptom reporting, automated triage, and timely clinical response, with the aim of delivering proactive, patient-centered care within a community oncology setting.

In this article, we report a qualitative evaluation of the RESPONSe program implementation that highlights the complexities of translating remote symptom monitoring from pilot innovation into routine cancer care delivery. Although quantitative evaluations can assess clinical outcomes and program utilization, qualitative methods are uniquely suited to understanding stakeholder experiences, implementation challenges, contextual factors, and mechanisms that influence program success. By exploring the perspectives of individuals involved in the implementation and operationalization of RESPONSe, this study seeks to provide practical insights into the barriers and facilitators associated with integrating ePRO-based remote symptom monitoring into routine oncology practice. These findings may inform future implementation efforts and contribute to the growing body of evidence on the sustainable adoption of digital symptom monitoring interventions within cancer care.

## 2. Methods

### 2.1. Design

This project was conducted as a qualitative post-implementation process evaluation of the RESPONSe program at Richmond Cancer Clinic (RCC) in Richmond, BC, Canada. It was designed as a qualitative descriptive study [16] and analyzed using deductive–inductive thematic analysis [17], with the aim of examining implementation experiences and identifying perceived barriers and facilitators associated with implementing a remote symptom monitoring program and transitioning from pilot program to operationalization.

A qualitative design was selected because the evaluation question was explanatory rather than comparative, concerned with how and why the program proceeded as it did within a specific organizational setting. Qualitative descriptive design [16] is a methodologically robust framework frequently employed within health services and implementation science research to examine complex clinical phenomena. Quantitative program metrics, including enrolment, survey completion, alert volumes, and acute care utilization, are collected routinely and have been reported separately [15]. In this evaluation, operationalization refers to the transition from a time-limited pilot to a routinely delivered clinical service, characterized by integration into clinic workflows, recurrent base-budget funding, and delivery as a standard component of care. It does not imply full technical integration with the institutional electronic medical record.

The evaluation was deliberately scoped to the perspectives of those responsible for designing, implementing, and operating the program. Patient experience has been assessed separately through experience surveys and a qualitative evaluation conducted in November 2025; early results have been reported previously [14], and the full findings were summarized in an internal report that informed program refinement. Patient involvement across the program lifecycle is described in Section 2.4.

Reporting follows the Consolidated Criteria for Reporting Qualitative Research (COREQ) [18], and a completed checklist is provided as Supplementary File S1.

### 2.2. Setting and Context

RCC is a community-based outpatient oncology clinic operating within British Columbia’s publicly funded provincial healthcare system as a site of the provincial Community Oncology Network (CON). Community oncology accounts for a substantial share of cancer treatment in the province: in 2021, CON sites delivered approximately 50% of all systemic therapy in British Columbia [19]. RCC also serves a linguistically diverse population. In the 2021 Census, 44.5% of Richmond residents reported a Chinese language as their mother tongue, with Cantonese and Mandarin represented in approximately equal proportions [20]. This demographic profile shaped several of the program design decisions described below, including the subsequent addition of a Traditional Chinese language version of the platform.

RESPONSe is an ePRO-based RSM program designed to support systematic symptom data collection and clinical triage for patients receiving chemotherapy [14]. The program originated as a clinic-led initiative rather than through an institution-wide mandate or centrally coordinated digital-health program. It was developed through a multi-phase, stakeholder-informed process emphasizing feasibility, workflow integration, and equitable access, and combines digital symptom monitoring with a dedicated symptom management nurse (SMN) role responsible for reviewing and responding to patient reports.

Of 545 patients initiating systemic therapy at RCC between January 2024 and June 2026, 381 began chemotherapy or antibody–drug conjugates therapy, of whom 271 (71%) were enrolled. The program was offered to all patients initiating these systemic treatments at any disease stage and site, provided they had access to home internet or a mobile device and could complete surveys in English or Chinese, either independently or with the help of a family member. Median age of participants was 66.5 years (range 20–92 years). Patients receiving immune-checkpoint inhibitors were excluded because the program’s symptom triage logic was developed around cytotoxic toxicity profiles and had not yet been adapted for immune-related adverse events. Among patients approached for enrolment, 29% did not participate. Reported reasons included lack of internet or device access, language barriers, and feeling overwhelmed by their medical circumstances.

At the time of writing, RESPONSe had been embedded in routine outpatient oncology care for approximately 30 months. In April 2025, the program transitioned from time-limited grant funding to recurrent operational funding, and is now delivered as a standard component of outpatient chemotherapy care at RCC.

### 2.3. Program Features

*Patient interface and support:* RESPONSe is delivered through SeamlessMD, a commercially available digital platform selected through competitive procurement and subsequently configured to the clinical and operational requirements of outpatient oncology care [14]. The platform operates separately from the health authority electronic medical record (EMR), without automated exchange of patient-level symptom data: clinicians access the dashboard through a separate login, and the SMN manually documents clinically relevant assessments and actions in the EMR.

Patients initiating intravenous systemic therapy are monitored over the first 6 months of treatment. Surveys, known to patients as Health Checks, are scheduled at a baseline visit, on days 3 and 10 following chemotherapy administration when symptom burden is expected to peak, and before scheduled clinic appointments so that patient concerns are available for physician review. Patients may also complete an optional Health Check at any time [15]. Reminders of a scheduled survey are sent by email and text message. Surveys are accessible via web browser or mobile application and are available in English and Traditional Chinese.

Following an initial self-reported 0–10 severity rating for symptoms from a predefined list of common chemotherapy-related side effects including those from the Edmonton Symptom Assessment System-revised (ESAS-r), patients are guided through symptom-specific question branches that elicit clinically actionable detail such as onset, frequency, severity modifiers, associated features, and functional impact. These adaptive pathways were derived from the pan-Canadian Oncology Symptom Triage and Remote Support (COSTaRS) practice guides and were developed iteratively with clinicians across successive design cycles. Free-text entry and photograph upload allow patients to describe symptoms that fall outside the structured categories.

*Triage and escalation:* RESPONSe was designed to function as clinical decision support rather than as an autonomous triage system. Survey responses are processed by symptom-specific conditional logic that classifies each report as mild, moderate, or severe, and every submission returns immediate self-management guidance and links to a patient education library. Moderate and severe reports generate alerts on a secure, web-based clinician-facing dashboard, which the SMN reviews during clinic hours. Moderate symptoms prompt follow-up within two working days, and severe symptoms are escalated urgently according to predefined clinical pathways. The SMN conducts patient outreach and coordinates escalation with the oncology provider. The basis for each classification is visible to the reviewing nurse, and the platform does not diagnose, prescribe, or modify treatment. Every clinical action follows from the assessment of the SMN or the oncologist rather than from the software output itself. The SMN records the clinical assessment in the patient’s chart and captures process data within the platform, including alert timing and time to response, for program monitoring purposes.

*Privacy and governance:* Prior to deployment, the platform underwent a privacy impact assessment and formal institutional review addressing privacy, security, information governance, and compliance. That review characterized the platform as a tool for collecting patient-reported outcomes, delivering educational content, and supporting clinician monitoring and follow-up, with clinical decisions remaining the responsibility of the care team.

### 2.4. Program Development and Implementation Milestones

**Figure 1:**
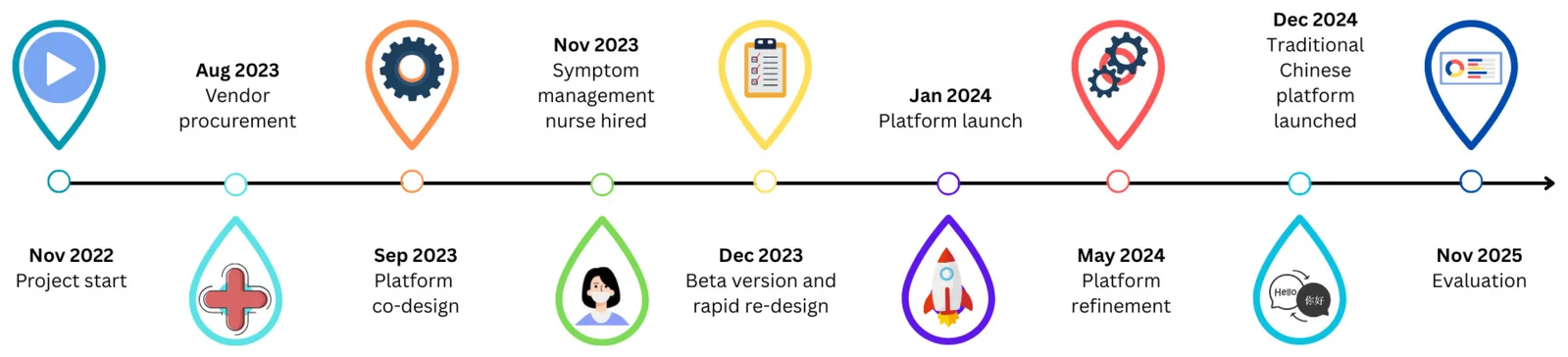
Key development and implementation milestones of RESPONSe program (2022–2025).

RESPONSe was developed in five phases between 2022 and 2025, summarized in Figure 1.

*Scoping and stakeholder engagement (2022):* Development began in partnership with the institutional virtual health team, with structured engagement at RCC involving oncologists, oncology nurses, operational leaders, and patient advisors. This phase defined program objectives, scope, and workflow requirements, informed by earlier patient interviews that had identified unmet needs for timely, proactive symptom support.

*Vendor selection (November 2022 to August 2023):* SeamlessMD was selected through competitive procurement on the basis of its configurable pathways, usability, and dashboard-based monitoring, which allowed oversight of a large patient cohort with triage rules and alert thresholds defined by the clinical team [14].

*Build and co-design (September to December 2023):* Successive design cycles involving nursing, virtual health, and the vendor produced the branching survey logic, COSTaRS-derived symptom questionnaires, automated triage flags, self-management content, and the educational resource library. In parallel, the SMN role was established and its clinical workflows developed, including in-person patient enrolment, dashboard monitoring, and documentation into the clinical record.

*Simulation and readiness testing (December 2023 to January 2024):* Full workflow simulations using demonstration accounts were run to test system reliability, refine escalation processes, and prepare clinical teams before go-live. Patients took part in this dry-run testing, and their feedback on content and usability was incorporated before launch.

*Launch and iterative refinement (January 2024 onward):* The program entered active implementation in January 2024. Patient feedback gathered through experience surveys and direct contact with the SMN informed subsequent development, including a refinement cycle in May 2024 and the addition of a Traditional Chinese language version in December 2024. The Chinese version was intentionally developed only after the main English program had gone through one iteration to refine survey and workflow issues. A qualitative patient experience evaluation was conducted in November 2025, with a final internal report used to inform program refinement to improve usability.

### 2.5. Participant Recruitment

Participants were sampled purposively along two dimensions: professional role (oncology physician, oncology nursing, virtual health, and operational leadership) and phase of program involvement (conceptualization, design and build, launch, and ongoing operations). The aim was to include at least one participant for each role in each phase in which that role was active, so that strategic decisions, workflow adaptations, leadership engagement, and equity considerations could be examined across the program lifecycle rather than at a single point in time.

A total of 10 staff met these criteria and were invited by email, with one physician declining due to time constraints and another physician did not respond to email invitations. The remaining eight consented and participated. The final sample comprised one oncologist, three nurses, two virtual health staff (one clinical planner and one leader), and two administrators (one operations director and one manager). It represented the full complement of staff with direct involvement in designing, implementing, or operating RESPONSe at the time of recruitment. All participants had been involved for one to three years, providing a longitudinal perspective on the program’s evolution. Verbal informed consent was obtained at the start of each interview.

Sample size adequacy was considered in terms of information power rather than data saturation [21]. Adequacy at this sample size is supported by the narrow and clearly bounded aim of the evaluation; a sample of high specificity, comprising individuals who personally designed and delivered the program; an applied analytic frame of implementation barriers and facilitators; interviews that yielded detailed, instance-specific accounts; and single-case analysis focused on one program rather than comparison across settings. Because the sample included every individual with direct program involvement, further recruitment was not available within the scope of this evaluation.

### 2.6. Data Collection

Development of the interview guide was informed by the framework for the staged evaluation of complex innovations [22]. These were organized around four areas: the participant’s role in the program and how it changed over time, difficulties encountered during design, launch, and ongoing operation, the adaptations made in response, and what they would do differently. Participants were asked throughout for specific instances rather than general impressions. The interviewer (MJC) piloted the guide with one research assistant (NAS) and minor revisions were made before interviews began. The full guide is provided as Supplementary File S2.

Individual interviews conducted by the research assistant (MJC) lasted 30–60 min. Interviews were conducted privately, with no other individuals present, via Zoom (Version 6.3.11) between May and August 2025, and were audio-recorded following verbal consent obtained at the start of each interview. MJC also recorded field notes during and after the interview to document observations and reflections. At that point the program had been operating for 16 to 19 months, and its transition to recurrent operational funding had occurred shortly before. Three participants took part in a brief second interview to clarify points raised in the first interview; one participant subsequently provided an additional paragraph by email to further elaborate on a prior response.

Recordings were auto-transcribed in Zoom and checked against the audio by MC, who corrected transcription errors. Transcripts were de-identified, accessible only to MC and JH, and imported into NVivo (Version 14.24.2). Transcripts were not returned to participants for review.

### 2.7. Reflexivity

All interviews and data analysis were carried out by MJC, a female research assistant with a master’s degree and training in qualitative research methods. The research assistant had an existing professional relationship with the physician lead and one nurse but was unfamiliar with the remaining six participants; therefore, she had a baseline knowledge of the program but was not involved in delivering the program. Participants were aware of the research team’s goals of identifying barriers, facilitators, and lessons for improvement, which would inform program development and knowledge dissemination.

### 2.8. Data Analysis

Transcripts were analyzed using deductive–inductive thematic analysis, combining an a priori organizing framework with inductively generated codes [17]. Analysis followed the phases described by Braun and Clarke: familiarization, code generation, searching for and reviewing themes, and defining and naming them [23].

MJC read each transcript in full alongside the audio recording and subsequently coding was conducted in NVivo. Implementation barriers and facilitators formed the deductive organizing frame, reflecting the aim of the evaluation, while the codes describing what those barriers and facilitators consisted of were generated inductively from participant accounts rather than specified in advance.

MJC developed an initial codebook based on a review of data, and resulting codes and data excerpts were compiled into a coding table. Coding was not independently duplicated, and this is acknowledged in the Section 6. The broader research team then reviewed this consolidated table of codes and excerpts and engaged in collaborative discussion to identify patterns, relationships, and areas of convergence and divergence across the data. Related codes were grouped and refined to develop overarching themes, corresponding to the technological, human, and organizational domains of implementation. The theme structure was reviewed by all authors, and theme and sub-theme labels were revised for consistency with the supporting data. Illustrative quotations were selected to represent the range of accounts contributing to each sub-theme. Two rounds of member checking with participants and additional staff were undertaken, with minor phrasing changes for precision and clarity.

## 3. Results

Three overarching themes reflected the key areas through which implementation challenges and adaptations unfolded: (1) tailoring technology for clinical fit, capturing the iterative work required to adapt a generic commercial platform to the specialized demands of oncology symptom monitoring; (2) designing care around patient and clinician users, encompassing relational, workforce, and equity considerations shaping adoption and sustainability; and (3) managing up towards operationalization, reflecting the organizational navigation required to secure leadership buy-in, funding, and institutional support for a frontline, clinic-led program. Together, these themes indicate that operationalization was a sociotechnical process in which technology adaptation, dedicated clinical ownership, evolving workflows, and organizational advocacy were interdependent.

### 3.1. Tailoring Technology for Clinical Fit

Across interviews, participants described challenges involved in adapting technology to oncology-specific clinical, operational, and patient needs. Three main technology-related sub-themes emerged: (1) system design and workflow integration, (2) signal processing and clinical relevance, and (3) user interaction and engagement (Table 1).

Most participants described systematic misalignment between generic commercial platform architecture and the specialized requirements of oncology symptom monitoring. The mismatch surfaced immediately:

“We ran into roadblocks right away because it just can’t do some of the logic that we wanted. […] So then you have to go back and adjust what you want to do… Sometimes we’ll have to come up with another way where we can achieve that goal. So there’s always limitations.”

(Virtual Health)

Closing that gap required substantial clinician-led customization of dashboards, surveys, and alert logic, and a willingness to treat vendor-stated limits as negotiable rather than fixed:

“It’s really important not to accept a limitation of the technology, but to push through it and challenge them and say ‘why not?’”

(Physician)

Ongoing optimization required iterative co-calibration with the vendor to balance competing priorities that became apparent during real-world use, including standardization versus patient self-expression, alert sensitivity versus clinical burden, and automation versus patient intent.

While use of a commercial platform enabled rapid implementation, it also introduced trade-offs in interoperability, contributing to workflow inefficiencies and fragmented data systems that limited program-level evaluation. For clinicians, the cost was felt in daily documentation:

“[RESPONSe] is not integrated with [the institutional EMR]. […] They have to go between a couple of different systems to document and find information. That […] doesn’t make it a seamless work process.”

(Virtual Health)

While interoperability was felt to be desirable from the outset, this was unattainable without a formal institutional mandate. Key functions such as a clinician-facing dashboard, patient survey submission, and embedded triage logic were not even on the organizational information technology roadmap, and requests to pursue them would have had no clear route forward. Only after the program had demonstrated patient and operational value, and planning for expansion to other sites had begun, did organizational discussions become possible regarding one-way transfer of selected ePRO data from the external platform into the EMR and development of an embedded visualization accessible within the existing clinical workflow. The technical scope and feasibility remained under development during the study period.

### 3.2. Designing Care Around Patient and Clinician Users

In contrast to the technological challenges of adapting a digital platform, several participants noted that successful implementation also depended on careful consideration of the user experience of the tools by both patients and clinicians. Two interrelated sub-themes were identified: (1) human factors influencing adoption and (2) workforce and workflow capacity (Table 2).

Across most interviews, the SMN role was described as foundational. Beyond coordinating workflows and triaging alerts, the SMN role functioned as a relational anchor for patients, positioning the digital platform as an extension of trusted care and thereby enhancing uptake and sustained engagement. Participants located the program’s value in the person rather than the technology:

“The program itself […] is really nothing without the actual human that returns the phone calls and manages all of [the alerts]. […] We really had to shift to the importance of the actual nurse.”

(Administrator)

Most participants also highlighted the importance of flexible and culturally-responsive features in supporting equitable participation, particularly caregiver enrollment pathways and Traditional Chinese translation, which helped address barriers related to language proficiency and digital literacy. Caregiver enrolment drew on a motivation already present:

“Caregivers usually are often very eager to participate because they feel that they can help.”

(Nurse)

Giving caregivers a defined role converted that willingness into a practical access pathway for patients facing language or digital-literacy barriers.

Most participants observed that the introduction of a new care model generated workflows and operational demands that strained existing capacity. While paper charting remained in use, information was recorded in the platform and again on the paper chart:

“We’re documenting all that [data on RESPONSe]. Plus, on top of that, we’re documenting […] in the patient’s chart, so it’s a lot of duplication. That’s a big challenge with the data collection.”

(Nurse)

This burden eased with the transition to a fully digital patient record. Recording process data such as alert timing and time to response remained an additional demand, however, and one that fed the program metrics later used to demonstrate the program’s value to leadership.

Across both sub-themes, the requirements participants described were human rather than technical, and they continued to evolve with each iteration of the program. Given the many ePRO platforms now available, this suggests no platform can be selected “correctly” at procurement; what can be selected is the capacity to adapt. That capacity must exist both in the technology, through configurability and a vendor committed to ongoing co-design and in the surrounding model of care, which must be able to keep translating change into practice.

### 3.3. Managing up Towards Operationalization

Organizational factors underscored the importance of “managing up”, a term from organizational management referring to the deliberate effort by frontline workers to proactively engage and build relationships with executives and operational stakeholders. Throughout implementation, the need to navigate institutional structures, leadership priorities, infrastructure constraints and funding uncertainty to sustain a frontline, clinician-led innovation was noted. Three sub-themes were identified: (1) organizational context and capacity; (2) strategic positioning and sustainability; and (3) implementation approach (Table 3).

Most participants described challenges in the gap between what the institution could support and what the clinic needed. The health authority EMR could not accommodate the program, and no formal mandate existed to require that it do so, so the team built alongside it, drawing on informal contacts in patient experience, decision support, and language services rather than on an assigned project structure.

While engagement and partnership were well resourced during planning and build, several participants described the organizational work as most intensive after launch, and particularly when sustainment was being sought. Grant funding had established the program but did not carry its running costs:

“Often you can get grants to start these kinds of things, but then it’s not part of your operational funding, so then you have to secure it. […] Even the cost of [the platform] every year is not something that is built into my budget.”

(Administrator)

As the initial funding approached its end date, effort shifted toward presenting program data to senior leadership, including enrolment and engagement metrics, patient experience results, and internal analyses of acute care utilization, in order to build the case for continuation and operationalization.

Participants described securing organizational support as a matter of reaching the right level of seniority with the right kind of evidence:

“You would want to get buy-in from the most senior leaders that you can. What is the data that helps you add a dollar value to the work that you’re doing?”

(Administrator)

Applying for institutional, regional, and provincial awards was one strategy for sustaining visibility, and success in these competitions provided external recognition that reinforced the program’s standing with leadership. Visibility and funding were described as closely linked:

“When more people know about it and the good work that’s being done, it’s easier to secure funding.”

(Administrator)

The organizational work described in this theme ultimately achieved its objective. In April 2025, RESPONSe transitioned from time-limited grant funding to recurrent operational funding within the health authority’s base budget and is now delivered as a standard component of outpatient chemotherapy care. Participants’ accounts of funding uncertainty therefore describe a period that has since been resolved, though the effort required to reach that point is the substance of this theme.

## 4. Discussion

Despite strong evidence that RSM with ePROs improves early symptom detection, patient–provider communication, quality of life, and acute care utilization, and despite endorsement from professional bodies such as American Society of Clinical Oncology, real-world adoption remains limited, particularly in community oncology and within the Canadian healthcare context [6,7,10,11,12,13,24,25]. Many promising digital health pilots fail to move beyond the pilot phase, creating the situation of having “perpetual pilots” where innovations that demonstrate feasibility never achieve sustained operationalization within routine care [26]. This qualitative evaluation addresses this gap by describing the implementation and operationalization of an RSM program in a community cancer clinic using a commercially available platform, a clinician-led approach, and patient-owned devices, and by deriving lessons for other community settings. Success depended on the interaction of an adaptable technology, dedicated clinical ownership, continuously evolving workflows, and sustained organizational advocacy. Of these, the organizational conditions proved hardest to move; technical limitations were largely resolvable through configuration, whereas interoperability, staffing, and continuation depended on institutional decisions the clinic could influence but not make.

Successful RSM programs have been established internationally, but often through funded research programs, multi-site trial networks, or centrally coordinated institutional initiatives. In the United Kingdom, eRAPID combined online symptom reporting with automated severity-dependent patient advice and direct integration into the hospital electronic patient record [27]. The European eSMART trial tested the Advanced Symptom Management System (ASyMS) in 829 patients across 12 cancer centers in five countries, using study-supplied handsets and thermometers [28]. In Australia, PROMPT-Care was embedded within the existing oncology information system across four public hospitals [29]. The US PRO-TECT cluster-randomized trial reached community oncology practices, but did so with central academic coordination, standardized workflows, and dedicated trial infrastructure [9]. Canadian examples, including the BC Cancer Kelowna immunotherapy pilot and the BC Cancer Victoria concurrent chemoradiation program, likewise reflect centralized models tied to specific treatment courses and dedicated hardware [24,25].

Despite their differences in geography and health system, these programs share a common set of enabling conditions: purpose-built software, formal integration with the institutional electronic record, dedicated research or program funding, defined eligibility criteria and time-limited study periods, and in several cases, hardware supplied to participants. In contrast, RESPONSe was implemented at a single community outpatient clinic without a formal institutional mandate, using a configured off-the-shelf commercial platform that operated alongside rather than within the health authority electronic medical record, delivered on patients’ own devices, and sustained as routine service delivery rather than as a funded study with a defined endpoint. The demands that followed were correspondingly different: configuring a generic platform for oncology triage, operating alongside rather than within the institutional record, and sustaining organizational attention without a defined endpoint. It is this work, rather than the conditions under which RSM has already been shown to succeed, that the present evaluation sets out to characterize.

The RESPONSe experience offers an example of what an RSM implementation can look like when it emerges primarily from the frontline, rather than from top-down institutional direction. Unlike centralized, institutionally-driven remote monitoring models built on custom infrastructure, RESPONSe evolved through local clinical leadership, iterative co-design with a commercial vendor, and pragmatic adaptation of existing tools. Institutionally led solutions offer advantages in standardization and interoperability but are often constrained by long development timelines, competing priorities, and limited flexibility [30]. A clinic-led commercial-platform-based approach trades some standardization for greater speed, local tailoring, and responsiveness to frontline needs, which may be particularly valuable in community settings where institutional infrastructure evolves more slowly. That RESPONSe achieved sustained operationalization without an initial enterprise-wide mandate suggests that institutional scale alone does not determine implementation success. The supports that eventually sustained the program, including dedicated clinical staffing, cross-functional institutional partners, external funding, and organizational commitment, were not in place at the outset. Each was assembled by the local team over the course of implementation, in most cases only after the program had begun to demonstrate value. This ordering is the substantive difference from programs in which those conditions are provided as a precondition of launch. In this evaluation, operationalization did not require full technical integration with the institutional EMR. EMR-based access to selected ePRO data remains a proposed optimization intended to streamline workflows, improve ePRO visibility, reduce duplicate documentation and improve physician engagement, rather than a prerequisite that had been achieved during the period studied.

Notably, many of the principles that emerged through this frontline-initiated process mirror those identified by established implementation frameworks. Although the RESPONSe program was designed before the publication of Basch et al.’s tenets for ePRO-based remote symptom monitoring, many of the same principles, including attention to software functionality, ePRO selection, alert logic, staffing, patient engagement, and equity, emerged independently through the implementation process [31]. This convergence suggests that these considerations reflect core operational requirements of RSM design, and that teams arriving at similar conclusions through different implementation pathways may be responding to common demands of this care model. While broad frameworks such as the PROTEUS guidelines offer valuable high-level direction, the RESPONSe experience illustrates the realities of operationalizing these principles within the constraints of a small community setting, translating them from recommendation to practice through iterative adaptation, frontline leadership, and sustained organizational navigation [32].

The centrality of a dedicated nursing role is the clearest example of this convergence. The need for defined staffing to review, triage and act on incoming symptom reports is well established in the ePRO implementation literature, appearing both in Basch et al.’s implementation tenets and in the perspectives reported by community oncology practices participating in the Alliance AFT-39 trial [8,31]. Trials such as eRAPID likewise relied on nursing staff to monitor incoming symptom alerts rather than on automated escalation alone [27]. Our findings add evidence that the same requirement holds in a community clinic, where the role had to be created, justified and defended rather than resourced as part of a trial protocol. In this setting the dedicated role was not merely a facilitator of implementation but a precondition for it.

Together, these observations translate into a set of practical lessons that may help other institutions navigate the gap between RSM evidence and routine implementation.

## 5. Key Lessons and Transferable Implementation Strategies

Several practical lessons emerged from operationalizing RESPONSe that may inform other community programs adopting RSM using a commercial platform. We summarize these as five transferable implementation strategies (Table 4). To make them usable outside our own setting, we have mapped each strategy to the corresponding domain of the Consolidated Framework for Implementation Research (CFIR) [33]. This mapping was performed post hoc to support transferability and comparison with other implementation reports. CFIR was not used as an a priori coding framework in our analysis, and the strategies below are derived from participant accounts rather than from the framework itself.

Underlying all five strategies was the core design principle that the technology should serve as a bridge for human connection and remain secondary to the clinical relationship. In our experience, sustained uptake did not follow from platform capability. It followed from the symptom management nurse, who introduced the program in person at treatment initiation and whose availability positioned the digital tool as an extension of care that patients already trusted rather than as a substitute for it. Programs that approach ePRO implementation primarily as a technical deployment risk optimizing the components of the system that matter least to the people using it. The strategies in Table 4 are offered in that light, as means of protecting clinical relationships under the operational demands of remote symptom monitoring rather than as ends in themselves.

## 6. Limitations

As this evaluation reports one clinic’s experiences, findings should be generalized to the conditions of a single community clinic operating within a publicly funded provincial system within an established community oncology network, serving a large proportion of Chinese-speaking patients.

All participants had been involved in designing or delivering the program, so staff who did not engage with it, or who had since left, are unrepresented. Two invited oncologists declined, both having made limited clinical use of the program; the sample therefore includes one medical oncologist and no non-adopters. Limited physician engagement was itself a finding, described here by those who observed it rather than those who were not involved. Accounts of development and launch were also retrospective, in some cases by three years.

Interviews were conducted by a member of the research team and several authors held leadership roles in the program, which may have discouraged participants from voicing criticism; coding was performed by a single researcher, rather than independently duplicated.

Several questions are out of scope of this evaluation, including patient perspectives which are examined separately for quality improvement purposes but have not been reported. At the point of reporting, the RESPONSe program was limited to cytotoxic chemotherapy and antibody–drug conjugates, while adaptations for immune-checkpoint inhibitor therapy are being developed and a bispecific therapy pathway is being planned. Finally, we did not compare staffing workload or costs against the usual level of care, and sustainability under future institutional leadership priorities remains uncertain.

## 7. Conclusions

This evaluation of the RESPONSe program highlights the complexities of operationalizing remote symptom monitoring using commercially available technology within a community oncology practice. The findings suggest that sustainable implementation depends on viewing RSM as a sociotechnical model of care that requires iterative refinement and organizational support to enhance therapeutic relationships. These findings carry implications on several levels. For practice, our experience suggests that community clinics may be able to begin developing RSM without a formal institutional mandate or full electronic-record integration, provided they secure dedicated clinical ownership, establish safe workflows alongside existing systems, and engage the organizational partners needed to support implementation and sustainability. For policy, if remote symptom monitoring is to become routine rather than exceptional in the community settings where much of systemic therapy is delivered [20], health systems will need to resource the dedicated clinical roles on which such programs depend, allow sufficient runway for value to be demonstrated, and create a clearer route from demonstrated value to operational funding. For research, the five strategies proposed here are testable propositions rather than conclusions: whether they transfer across community sites and how their costs compare with usual care are questions this evaluation opens rather than closes. The transition of RESPONSe from pilot to funded clinical service illustrates how this gap may be closed through iterative adaptation, dedicated clinical ownership, and sustained partnership between frontline teams and organizational leaders.

## Figures and Tables

**Table 1:** Technology-related factors influencing RSM implementation.

Theme	Barrier	Facilitator
System Design and Workflow Integration		
Customizing generic platform architecture with oncology-specific clinical workflows	Default, off-the-shelf dashboard design did not align with oncology symptom monitoring workflows, resulting in a cluttered, non-specialized interface where key clinical information was buried, hindering efficient use and failing to reflect workflow priorities.	Iterative, clinician-driven dashboard refinement including removal of non-essential fields, repurposing of data elements, highlighting key information, and incorporation of continuous feedback, improved usability and alignment with clinical needs.
Using a commercial platform resulted in trade-offs between rapid implementation and interoperability	Operating across a standalone RSM ** platform and a separate EMR * required multiple logins and manual data transfer, increasing cognitive burden and workflow inefficiency.	Establishing clear workflows mitigated immediate inefficiencies, while demonstrated clinical utility enabled engagement with organizational stakeholders to advance discussions on organizational discussions regarding one-way transfer and EMR-based display of selected ePRO *** data.
Bridging domain expertise gaps through iterative co-design	Limited vendor subject-matter expertise in oncology symptom management and limited clinician understanding of platform capabilities led to misalignment in initial system design and required repeated clarification of clinical workflows and expectations.	Ongoing vendor engagement enabled iterative co-design, progressively aligning platform functionality with clinical requirements.
Fragmented data ecosystems constraining program-level insight	Disparate data streams and organizational silos necessitated repeated data requests and manual aggregation, limiting ability to generate a cohesive, real-time view of program performance.	Development of structured data collection workflows and movement toward a centralized, regularly updated dashboard to enable more efficient aggregation for program evaluation and iterative refinement.
Challenging perceived limits through sustained vendor engagement	Fragmentation across vendor roles created a translation gap, with clinical requirements misinterpreted or prematurely dismissed, resulting in inconsistent assessments of feasibility. Limited transparency regarding platform capabilities further complicated determination of whether constraints were technical, operational, or communication-related.	Willingness to revisit and challenge initial constraints helped to convert perceived limitations into negotiable design space.
Signal Processing and Clinical Relevance		
Calibration of alert sensitivity to optimize clinical signal	Misaligned alert thresholds (overly sensitive or insufficiently responsive) undermined actionability and increased nursing workload through notification fatigue.	Frequent feedback loops with high-level nursing engagement enabled rapid, iterative adjustment of alert thresholds, aligning system sensitivity with clinical context and improving the relevance of alerts.
Misalignment between alert generation and patient care-seeking intent	Routine symptom reporting generated alerts independent of patients’ need for clinical follow-up, including for symptoms that had resolved or were self-managed, resulting in unnecessary nursing calls and increased workload.	Introducing a patient-directed option within surveys to decline a nursing call when self-managing aligned alerts with care needs, reducing unnecessary follow-up while preserving appropriate escalation.
User Interaction and Engagement		
Patient interface design ambiguity leading to unintended patient use and data inconsistencies	Unclear or unintuitive interface design created patient confusion, leading to atypical survey submission behavior and data inconsistencies.	Active clinician monitoring to detect anomalies, combined with a responsive vendor capable of investigating usage patterns and implementing interface redesign, enabled timely correction.
Tension between standardization and personalization in symptom reporting metrics	Standardized survey design to support triage logic created a mismatch between clinician-defined data needs and patients’ desire to describe their experiences, with structured surveys perceived as lacking the granularity and nuance needed to accurately represent symptoms.	Iterative customization of survey design such as the addition of free-text fields and expansion of reportable symptoms, enabled more patient-centered expression while preserving core triage functionality.
Passive engagement driven by convenience at the expense of deeper platform adoption	Reminders with embedded links improved survey completion but promoted passive, prompt-driven use, limiting patients’ familiarity with the platform and reducing independent use of features such as ad hoc reporting and the educational library.	Embedding structured teaching at onboarding and at a defined mid-point acts as a corrective strategy, transitioning patients from passive compliance to more active, informed use of the platform’s full capabilities.

* EMR = electronic medical record. ** RSM = remote symptom monitoring, *** ePRO = electronic patient reported outcomes.

**Table 2:** Workforce, workflow, and patient-centered factors influencing RSM implementation.

Theme	Barrier	Facilitator
Human Factors Influencing Adoption		
Dedicated role ownership as a foundation for program operationalization	Absence of an existing dedicated symptom management nurse role, limiting ownership and coordination of program activities.	Creation of a dedicated SMN * role provided clear ownership, enabling active program championing, coordinated patient support, and adaptive workflow integration as the program evolved.
Embedding digital monitoring within trusted clinical relationships	During treatment initiation, patients can experience information overload and emotional burden, which may limit their capacity to engage with new digital tools.	Framing the RSM ** program as an extension of a trusted clinical relationship, with in-person onboarding by the SMN, provided a relational anchor that improved engagement and uptake.
Flexible and culturally-responsive design supports equitable patient participation	Limited English proficiency and variable digital literacy reducing accessibility and independent program use for some patients.	Flexible, culturally-responsive design such as multiple participation pathways including caregiver enrollment, web- and app-based access, automated reminders, and translation (Traditional Chinese version), reduced barriers and enhanced accessibility and engagement.
Managing uncertainty during adoption of a novel care model	Introduction of a new digital care model disrupted established workflows and roles, with initial staff uncertainty, knowledge gaps, and lack of established protocols or onboarding pathways creating discomfort and concerns regarding reliability, follow-up, and role clarity.	Structured change management through physician and nurse champions, early working group engagement, and deliberate knowledge transfer, supported staff adaptation, built confidence, and enabled smoother integration of the new care model.
**Workforce and Workflow Capacity**		
Aligning continuous patient needs with finite clinical capacity in always-on monitoring systems	Continuous, unpredictable patient symptom needs often occurring outside clinic hours and in clusters, can create demand that exceeds available nursing capacity. In particular, accumulated post-weekend alert volumes can compound stress beyond simple notification overload.	Adaptive strategies including adjustment of alert thresholds, incorporation of patient-directed options to decline nurse contact, embedding self-management resources and clear escalation pathways for after-hours care, and empowering nurses to apply clinical judgment in triaging alerts, helped align system demands with available capacity and reduce unnecessary workload.
Ensuring continuity of specialized nursing coverage in a limited workforce model	A limited pool of trained symptom management nurses created vulnerability to absences, leading to workload surges for covering staff and concerns about consistent service delivery.	Cross-training of additional relief nursing staff, alongside flexible work arrangements (e.g., remote coverage), contingency staffing plans, enabled more reliable coverage and improved continuity of care.
Physician bandwidth constraints in adopting new care technologies	High baseline clinical workload, coupled with the need to access separate platforms, interpret unfamiliar data formats, and engage outside routine workflows, limited physician engagement with the system.	Access to selected ePRO *** information within the existing EMR workflow through embedded visualization identified as a potential means of reducing cognitive and operational barriers to physician engagement.
Data fragmentation and increased documentation burden from hybrid paper-EMR workflows	Concurrent use of paper and digital systems limited real-time data accessibility and required duplicated documentation across platforms and media, increasing administrative burden and workflow complexity.	Transition to a fully digital EMR **** with elimination of paper charts improved data accessibility and reduced documentation burden by minimizing duplication and streamlining workflow processes.

* SMN = symptom management nurse, ** RSM = remote symptom monitoring, *** ePRO = electronic patient reported outcomes, **** EMR = electronic medical record.

**Table 3:** Organizational factors influencing RSM implementation.

Theme	Barrier	Facilitator
Organizational Context and Capacity		
Institutional infrastructure constraints shape the pace and form of digital innovation	Large health system EMRs * lacked flexibility to rapidly incorporate emerging digital tools. The program did not align with the existing organizational EMR * roadmap, limiting opportunities for interoperability and scalability within existing infrastructure.	Demonstration of unmet clinical needs not addressed by the institutional EMR * justified parallel implementation, enabling timely deployment while building a case for future integration.
Innovation in large organizations requires navigating informal networks and institutional capacity	A clinic-led initiative without a formal institutional mandate lacked dedicated resources and faced difficulty identifying appropriate organizational partners to support required work.	Leveraging informal networks, pursuing external funding opportunities, and engaging in cross-functional collaboration (e.g., patient experience, decision support/analytics, translation) enabled continued progress.
Strategic Positioning and Sustainability		
Sustaining institutional visibility amid leadership turnover	Frequent management turnover disrupted continuity, leading to gaps in leadership awareness, knowledge, shifting priorities, and uncertainty regarding sustained organizational support.	Ongoing engagement with senior leadership, use of legacy documentation, and proactive onboarding of new working group members maintained project visibility.
Tension between pilot funding timelines and need for program stability	Uncertainty about program continuation beyond the pilot phase undermined morale and limited sustained engagement, as teams delivered care without assurance of long-term continuity.	Pursuit of internal and external awards and grants, alongside active dissemination through presentations and organizational engagement, strengthened program visibility, demonstrated value, and built momentum toward sustainable funding.
Defining program value is an evolving process during implementation	Initial uncertainty around meaningful outcome metrics led to overinclusive data collection and increased administrative burden without clear evaluation focuses.	Continuous engagement of leadership through meetings and presentations helped refine key metrics over time, enabling more targeted data collection.
Implementation Approach		
Implementation required an adaptive quality improvement approach	Unanticipated challenges emerged during real-world use, due to vendor limitations and evolving clinical knowledge of patient preferences.	Continuous evaluation, patient feedback surveys, dry-run testing prior to go-live, regular vendor collaboration supported iterative refinement and supported a culture of quality improvement.
Institutional trust as a prerequisite for digital adoption	Concern about software privacy and data security created initial hesitation in adopting a third-party platform.	Pre-existing privacy impact assessments, formal institutional reviews, and endorsement from leadership about vendor compliance established trust and supported adoption.

* EMR = electronic medical record.

**Table 4:** Five transferable implementation strategies for community-based RSM, mapped to CFIR domains and construct definitions.

CFIR * Domain	CFIR Constructs	Implementation Strategy
Innovation	Adaptability	Select a vendor contractually committed to iterative co-design, not one promising customization at sale
Individuals	Innovation Deliverers	Establish a dedicated role with explicit program ownership and relational patient onboarding
Implementation Process	Assessing Needs Planning	Define organizational value and success metrics before launch
Implementation Process	Reflecting & Evaluating Adapting	Treat implementation as continuous quality improvement, not deployment
Inner Setting	Relational Connections Communications Funding	Sustaining leadership engagement as an ongoing activity, not a launch task

* CFIR = Consolidated Framework for Implementation Research.

## Data Availability

The interview transcripts generated and analyzed during this evaluation are not publicly available. The sample comprised eight individuals at a single clinic, several of whom hold unique organizational roles, and the transcripts therefore cannot be de-identified to a standard that would protect participant confidentiality. Participants provided verbal consent to take part in a quality improvement evaluation and did not consent to public deposition of their data. The interview guide and the completed COREQ checklist are provided as Supplementary File S1 with this article. The coding framework developed during analysis is available from the corresponding author on reasonable request.

## Supplementary Materials

The following supporting information can be downloaded at: https://www.mdpi.com/article/10.3390/curroncol33090561/s1, File S1: COREQ (COnsolidated criteria for REporting Qualitative research) Checklist; File S2: Interview Procedure.
