# Supplementary material for: Practical Insights from Operationalizing a Remote Symptom Monitoring Program Using a Commercial Platform in a Canadian Community Cancer Clinic: A Post-Implementation Qualitative Evaluation
Source: Curr Oncol. 2026 Sep 16;33(9):561. doi: 10.3390/curroncol33090561 (PMC13605967; doi:10.3390/curroncol33090561)
Supplement: Supplementary file 1 [file curroncol-33-00561-s001.zip › Supplementary File S2.pdf]

Interview Procedure

Pre-Interview Preparation
MC to send invitation email to participants, staggered over several weeks. MC to book Zoom meetings with participants, sending meeting links as Calendar invites with zoom links. MC to test recording function on Zoom and check transcripts.

Interview Timeline
The timeline of a typical interview.

**Time**	**Item**
0:00 - 0:05	Login, greetings, late buffer
0:05 - 0:08	Pre-interview brief, questions
0:00 - 0:50	Interview<br>● Aim for responses 5 minutes per question<br>● Aim for 7 - 12 questions, with/without follow-ups
0:50 - 1:00	Post-interview brief, questions goodbye, late buffer

Pre-interview Brief
General talking points for an interview brief to give to participants before the interview happens.

Pre-interview Brief Script

- Thank you for joining me today.
- What we are looking for:
    - The goal today is to gather your ideas on barriers and solutions regarding its implementation, and areas for improvement.
- How this will work:
    - This is a semi-structured interview lasting about 30 - 60 minutes.
    - I will ask some questions and you can answer in free form. There are no right or wrong answers, so you can reply with whatever feels most important to you. We want to hear your honest experiences and perspectives, whether positive or negative.
    - When you are describing an event, please list the date, project phase, or project version if possible.
    - If there is anything that I would like more information on, I may ask follow up questions.
    - If you are unclear on any information, feel free to clarify the question or ask me to repeat it.
- Consent:

○ Your participation is voluntary. You can stop the interview at any time. After the interview, if you change your mind, you can email me.<br>○ I will be recording the Zoom call. We will de-identify everything you say. We will use the Zoom AI I transcription service to put what you said into writing, but all your data will be de-identified.<br>○ We will store the interviews in a secure location. Only I and JH will review the information. Once the research is complete, we will delete the recordings.<br>○ Do I have your permission to record this interview? [Record it if yes]<br>● Questions<br>○ Any questions?

Interview questions are individualized according to the provider's background and timeline of involvement for each of 10 participants.

Participant 1	
Introduction	● Can you describe your overall experience with the RESPONSe program?<br>○ What is your role with the RESPONSe project?<br>○ How long have you been involved in the RESPONSe project?<br>○ What does your day-to-day look like?
Idea	● What were the key challenges or gaps in patient care that encouraged you to investigate the RESPONSe program?<br>○ How did you determine that these challenges required a technological solution rather than an alternative?<br>○ What were the biggest feasibility concerns early on and how were those concerns addressed in the planning phase? Eg. software function, patient engagement, clinical workload
Development	● What were the biggest challenges in designing and setting up the RSM system and how were those addressed?<br>○ Eg. workflows, funding, institutional buy in?<br>○ How did you address technical or logical constraints during this phase?<br>○ Were there any elements of the development phase that you would've done differently or recommend other groups to do?
Exploration	● What were the biggest challenges during the initial implementation of the RSM program?<br>○ Eg. provider buy-in, patient adoption, tech issues, equity

	○ What challenges do you observe related to integrating into practice?<br>○ Were there any strategies that were effective in overcoming the challenges you mentioned?<br>○ If you could do something different, what would you do to improve the implementation?
Assessment	● Were there any unexpected barriers that arose when evaluating the program's effectiveness? What did you do to overcome these barriers?<br>○ What were factors that improved clinical decision-making and patient management?<br>○ If you could repeat the process, what additional metrics or data would have been useful for assessing the program's success?
Long-term Follow up	● What are the biggest challenges in sustaining the RSM over the long term? What strategies did you use to overcome these barriers?<br>○ Looking at long-term sustainability, what do you think are some important lessons learned?
Conclusion	● Looking back at the entire implementation process, what was the most important lesson learned or take-away?<br>● Do you have any last thoughts or anything you'd like to add?

Participant 2	
Introduction	● Can you describe your overall experience with the RESPONSe program?<br>○ What is your role with the RESPONSe project?<br>○ How long have you been involved in the RESPONSe project?<br>○ What does your day-to-day look like?
Idea	● What were the key challenges or gaps in patient care that encouraged you to investigate the RESPONSe program?<br>○ How did you determine that these challenges required a technological solution rather than an alternative?<br>○ What were the biggest feasibility concerns early on and how were those concerns addressed in the planning phase? Eg. software function, patient engagement, clinical workload<br>● Talk about barriers or changes for implementation?
Development	● What were the biggest challenges in designing and setting up the RSM system and how were those addressed?<br>○ Eg. workflows, funding, institutional buy in?

	○ How did you address technical or logical constraints during this phase?<br>○ Were there any elements of the development phase that you would've done differently or recommend other groups to do?<br>● Can you tell me about bidding/challenging for vendours?
Exploration	● What were the biggest challenges during the initial implementation of the RSM program?<br>○ Eg. provider buy-in, patient adoption, tech issues, equity<br>○ What challenges do you observe related to integrating into practice?<br>○ Were there any strategies that were effective in overcoming the challenges you mentioned?<br>○ If you could do something different, what would you do to improve the implementation?
Assessment	● Were there any unexpected barriers that arose when evaluating the program's effectiveness? What did you do to overcome these barriers?<br>○ What were factors that improved clinical decision-making and patient management?<br>○ If you could repeat the process, what additional metrics or data would have been useful for assessing the program's success?
Long-term Follow up	● What are the biggest challenges in sustaining the RSM over the long term? What strategies did you use to overcome these barriers?<br>○ Looking at long-term sustainability, what do you think are some important lessons learned?<br>○ You talked about funding from the Ministry. Could you tell me if there are any barriers and workarounds?
Conclusion	● Looking back at the entire implementation process, what was the most important lesson learned or take-away?<br>● Do you have any last thoughts or anything you'd like to add?

Participant 3	
Introduction	● What is your role with the RESPONSe project?<br>○ Can you describe your overall experience with the RESPONSe program?<br>○ How long have you been involved in the RESPONSe project?<br>○ What does your day-to-day look like?

Idea	● What were the key challenges or gaps in patient care that encouraged you to investigate the RESPONSe program?<br>○ How did you determine that these challenges required a technological solution rather than an alternative?<br>○ What were the biggest feasibility concerns early on and how were those concerns addressed in the planning phase? Eg. software function, patient engagement, clinical workload<br>○ Other staff mentioned hosting dinner meetings to get clinician buy-in. What were challenges with buy-in? How can you encourage people to participate?<br>● Tell me about implementation concurrent with clinic move and Cerner?
Development	● What were the biggest challenges in designing and setting up the RSM system and how were those addressed?<br>○ Eg. workflows, funding, institutional buy in?<br>○ How did you address technical or logical constraints during this phase?<br>○ Were there any elements of the development phase that you would've done differently or recommend other groups to do?
Exploration	● What were the biggest challenges during the initial use of the RSM program?<br>○ Eg. provider buy-in, patient adoption, tech issues, equity<br>○ What challenges do you observe related to integrating into practice?<br>○ Were there any strategies that were effective in overcoming the challenges you mentioned?<br>○ If you could do something different, what would you do to improve the implementation?
Assessment	● Were there any unexpected barriers that arose when evaluating the program's effectiveness? What did you do to overcome these barriers?<br>○ What were factors that improved clinical decision-making and patient management?<br>○ If you could repeat the process, what additional metrics or data would have been useful for assessing the program's success?
Long-term Follow up	● What are the biggest challenges in sustaining the RSM over the long term? What strategies did you use to overcome these barriers?<br>○ Looking at long-term sustainability, what do you think are some important lessons learned?
Conclusion	● Looking back at the entire implementation process, what was the most important lesson learned or take-away?

	● Do you have any last thoughts or anything you'd like to add?

Participant 4	
Introduction	● Can you describe your overall experience with the RESPONSe program?<br>○ What is your role with the RESPONSe project?<br>○ How long have you been involved in the RESPONSe project?<br>○ What does your day-to-day look like?
Idea	● What were the key challenges or gaps in patient care that encouraged you to investigate the RESPONSe program?<br>○ How did you determine that these challenges required a technological solution rather than an alternative?<br>○ What were the biggest feasibility concerns early on and how were those concerns addressed in the planning phase? Eg. software function, patient engagement, clinical workload
Development	● What were the biggest challenges in designing and setting up the RSM system and how were those addressed?<br>○ Eg. workflows, funding, institutional buy in?<br>○ How did you address technical or logical constraints during this phase?<br>○ Were there any elements of the development phase that you would've done differently or recommend other groups to do?
Exploration	● What were the biggest challenges during the initial implementation of the RSM program?<br>○ Eg. provider buy-in, patient adoption, tech issues, equity<br>○ What challenges do you observe related to integrating into practice?<br>○ Were there any strategies that were effective in overcoming the challenges you mentioned?<br>○ If you could do something different, what would you do to improve the implementation?
Assessment	● Were there any unexpected barriers that arose when evaluating the program's effectiveness? What did you do to overcome these barriers?<br>○ What were factors that improved clinical decision-making and patient management?<br>○ If you could repeat the process, what additional metrics or data would have been useful for assessing the program's success?

Long-term Follow up	● What are the biggest challenges in sustaining the RSM over the long term? What strategies did you use to overcome these barriers?<br>○ Looking at long-term sustainability, what do you think are some important lessons learned?
Conclusion	● Looking back at the entire implementation process, what was the most important lesson learned or take-away?<br>● Do you have any last thoughts or anything you'd like to add?

Participant 5	
Introduction	● Can you describe your overall experience with the RESPONSe program?<br>○ What is your role with the RESPONSe project?<br>○ How long have you been involved in the RESPONSe project?<br>○ What does your day-to-day look like?
Exploration	● What were the biggest challenges during the initial implementation of the RSM program?<br>○ Eg. provider buy-in, patient adoption, tech issues, equity<br>○ What challenges do you observe related to integrating into practice?<br>○ Were there any strategies that were effective in overcoming the challenges you mentioned?<br>○ If you could do something different, what would you do to improve the implementation?<br>● Can you tell more translations and how that worked? How did that help?
Assessment	● Were there any unexpected barriers that arose when evaluating the program's effectiveness? What did you do to overcome these barriers?<br>○ What were factors that improved clinical decision-making and patient management?<br>○ If you could repeat the process, what additional metrics or data would have been useful for assessing the program's success?
Long-term Follow up	● What are the biggest challenges in sustaining the RSM over the long term? What strategies did you use to overcome these barriers?<br>○ Looking at long-term sustainability, what do you think are some important lessons learned?
Conclusion	● Looking back at the entire implementation process, what was

	the most important lesson learned or take-away?<br>● Do you have any last thoughts or anything you'd like to add?

Participant 6	
Introduction	● Can you describe your overall experience with the RESPONSe program?<br>○ What is your role with the RESPONSe project?<br>○ How long have you been involved in the RESPONSe project?<br>○ What does your day-to-day look like?
Exploration	● When you had first adopted the program, what initial challenges or uncertainties emerged? How did you address them?<br>○ What additional support/resources would have been needed to improve early engagement?<br>● When you were just starting to adopt the program, what was the learning process like?<br>● When patients were adopting the process, what did the process look like? Was there resistance or barriers?<br>● Talk to me about equity for elderly or lack of technology literacy. What was considered? What was implemented?
Assessment	● How did you assess the impact of the Response project during the early roll out?<br>○ What are specific indicators that you looked for (eg. patient feedback, time saving, outcomes)?<br>○ It seemed that you were collecting a lot of data on the project. What type of data was necessary? What happened to this data?<br>● In terms of data collection to evaluate the project, what data is useful to collect? What do you think about the volume of data collected?
Long-term Follow up	● From your perspective, how sustainable is this system in the long term? If there are any barriers, what would those be?<br>○ What would help improve sustainability of your role in this system?
Conclusion	● Looking back at the entire implementation process, what was the most important lesson learned or take-away?<br>● Do you have any last thoughts or anything you'd like to add?

Participant 7

Introduction	● Can you describe your overall experience with the RESPONSe program?<br>○ What is your role with the RESPONSe project?<br>○ How long have you been involved in the RESPONSe project?<br>○ What does your day-to-day look like?
Development	● What were the challenges associated with integrating the Response program into nursing workflow? How did you overcome them?<br>○ Did you have specific feedback/suggestions that were incorporated into the final design?
Exploration	● When you had first adopted the program, what initial challenges or uncertainties emerged? How did you address them?<br>○ What additional support/resources would have been needed to improve early engagement?
Assessment	● How did you assess the impact of the Response project during the early roll out?<br>○ What are specific indicators that you looked for (eg. patient feedback, time saving, outcomes)?
Long-term Follow up	● From your perspective, how sustainable is this system in the long term? If there are any barriers, what would those be?<br>○ What would help improve sustainability of your role in this system?
Conclusion	● Looking back at the entire implementation process, what was the most important lesson learned or take-away?<br>● Do you have any last thoughts or anything you'd like to add?

Participant 8	
Introduction	• Can you describe your overall role and experience with the RESPONSe program?<br>• How long have you been involved in the RESPONSe project?
Exploration	• When you were first exploring the implementation of the RESPONSe project, what were some of the main challenges you faced?<br>o How did you overcome them?<br>• Were there any challenges or barriers faced when working with the vendors?<br>• How did you assess financial feasibility or cost-benefit?<br>o Were there any key metrics or data that was used for these

	calculations/studies?<br>• Were there any barriers to support from leadership/governance?<br>o What worked well to gain organizational support/buy-in?
Long-term Follow up	• What were the biggest administrative or financial barriers to sustaining this project overtime?<br>o How did you overcome any barriers/gaps?<br>• Were there organizational concerns about sustainability or upfront costs?<br>• What are some strategies that can help teams overcome administrative/governance barriers?
Conclusion	• Looking back at the entire implementation process, what was the most important lesson learned or take-away?<br>• Do you have any last thoughts or anything you'd like to add?

Questions for Elaboration and Clarification
Some questions are present to ask for more information.

Questions for Elaboration/Clarification	
Questions for Elaboration	● Can you elaborate more on …?<br>● Can you explain what you mean by …?<br>● Can you give me an example of when that happened or might happen?<br>● Can you give me an example of what that looks like in your day to day?<br>● How did your experience of … compare to …?<br>● How did that make you feel?
Questions for clarification	● Would you say …?<br>● So what I'm hearing is… would you say that's correct?<br>● You said… How did you come to that decision?

Taking Notes During the Interview
During the interviews, MC will take notes on:

- Any documents, programs, or regulatory bodies to clarify with JH.
- Non-verbal cues (sighs, facial expressions, gestures, body language etc) with time stamp

Example interview template and notes below.

Participant #	#
Date	YYYY-MM-DD
Time (MM:SS)	Notes
Ex. 06:32	*Ex. Sighs and pauses before speaking*
12:10	*Uses hands to indicate roughly 1 foot long*
42:03	*Look up definition for “PROM”, patient mentioned “ABC survey”*

Master list of Abbreviations
During the interviews, MC will create a running master list of abbreviations: RESPONSe, ePRO, RSM, SMN, PROMs, VCH, etc. Example master list of abbreviations is below.

Abbreviation	Definition
RESPONSe	*REmote Symptom and Patient mONitoring System*
PROM	*Patient reported outcome measure*
PRO, ePRO	*(electronic) patient reported outcome*
RSM	*Remote symptom monitoring*
SMN	*Symptom management nurse*
…	*…*

Post-Interview Brief
Notes to close an interview.

Post-interview Brief
- I will stop the recording. [Stop Recording]
- Is there anything you’d like to say off the record?
- Thank you for sharing your thoughts today. It was really helpful to gather your ideas. We will share the results of our interviews later this year.
- Do you have any questions? If anything comes up, feel free to email me.

Post-Interview Thank You Email
Send this email to them within 24 hours of concluding the interview.

Subject: Thank You for your Interview Participation

Hi [Participant’s Name],

I would like to send a quick note to thank you for taking the time to participate in our interview. Your insights are incredibly valuable and I appreciate your willingness to share

your experiences.

If you have any additional thoughts, feel free to reach out by email. Thanks again!

Best regards,

[Research Assistant Name]

Second Interview

If, during any part of the data analysis process, the research team (MC, NS, JH) determines that further information from any participant is required, MC will reach out to the participant to ask for a second interview. If the participant is willing, MC will conduct a second interview with refined questions.
